# The Present and Future of the Poor-Law Infirmary

**Published:** 1913-04-19

**Authors:** C. Thackray Parsons

**Affiliations:** Medical Superintendent Fulham Infirmary.


### THE PRESENT AND FUTURE OF THE POOR-LAW INFIRMARY.

(*Continued from page 16.*)

By C. Thackray Parsons, M.D. Lond., Medical Superintendent Fulham Infirmary.

A study of the system under which accommodation for the institutional treatment of the sick poor in London is provided by the Poor Law leads one inevitably to the conclusion that the origin of nearly all its defects and the stumbling-blocks in the way of progress are want of co-ordination and central administration. It is true that the Local Government Board exercises a certain amount of control by means of its inspectors and has done and is doing excellent work in rousing Boards of Guardians to a sense of their responsibility, and that of recent years it has caused to be provided accommodation available for all the London parishes for certain special classes of patients. Nevertheless, for all practical purposes each infirmary is under completely separate management and has no relations whatever with its fellows. As a direct consequence the infirmaries exhibit, as we have seen, extraordinary variations in structure, equipment, staff, and general efficiency, and one finds one infirmary over-crowded whilst another a mile or two from it may be only partly filled. The difficulty in the way of the provision of a specialist staff arises from the same causes. The number of cases requiring the services of such a staff in any one infirmary is comparatively small, and is insufficient to lead even those Boards which recognise the need, to face the expense. It is indeed unfair to expect any one Board to initiate a change of this importance. The provision could only be made economically by a number of Boards acting in concert. But the difficulty of getting any such concerted action was well illustrated when an attempt was made a few years ago to induce all the Metropolitan Boards of Guardians to adopt a uniform system for the training and examination of their probationers. A scheme was prepared and 32 Boards were invited to send representatives to consider it. But, notwithstanding the admitted defects of the present want of uniformity, and, probably, partly in consequence of the known fact that some of the infirmaries were giving an insufficient training, only three Boards supported the scheme, which accordingly fell through. But, in addition to this lack of co-operation between the different infirmaries, they are curiously isolated from the general medical world. Great as are the potentialities of their clinical material, the Poor-Law infirmaries are not connected in any way with the medical schools, and most people regard them as occupied by patients for whom little is done or can be done. The Poor-Law infirmaries still suffer from the obloquy which is attached to the very words "Poor Law," and one constantly sees newspaper references and hears opinions expressed by the general public, both medical and lay, which show how widespread is the ignorance of the work done in them and how great is the aversion in which they are still held by many. The Poor-Law infirmary and the Poor-Law patient are looked upon as being upon an entirely different and much lower plane than the voluntary hospital and its inmates. For this ignorance and for this false opinion upon the part of the public, Boards of Guardians are themselves largely to blame. Their discussions often show a lack of dignity and inability on the part of many of their members to appreciate the needs of the sick. Such discussions are only too often fully reported and commented upon by the lay and medical press, whilst the real work which is being unobtrusively performed is apt to be entirely unreported. Only three or four of the London Boards of Guardians publish any account whatever of the medical work performed within their infirmaries, and the medical staff is usually too busily occupied in coping with the work to be done in the wards to devote time to collecting statistics and publishing the results obtained. It is only by personal inquiries at the different infirmaries that anyone interested can obtain information as to what is actually being done.

If there is to be any great advance in the efficiency of the Poor-Law infirmary in London, and especially if all the infirmaries are to be brought up to the same general standard they must all be brought under one central administrative body. Such a central administrative body could be found either in the present Metropolitan Asylums Board. or in the Public Health Committee, enlarged if necessary, of the London County Council. Under the ægis of such a body the connection of the infirmary in the public mind with the old evil associations of the Poor Law and pauperism would be lost. And as a result of the reforms which the enlargement of the area of administration would permit, a large number of beds would at once become available and could be used, if necessary, for the hospital treatment of insured persons. It is somewhat difficult to estimate how many beds would be set free for this purpose. Even under existing conditions, and in the busiest time of the year, there would appear to be about 1,000 empty beds in the London infirmaries. But when one remembers that from 18 to 36 per cent. of the present inmates belong to the senile class, that the average length of stay varies in different infirmaries from 32 to 112 days, whilst in voluntary hospitals it is only about 19 days, and that the present staff is insufficient to do the work with the maximum efficiency, one cannot doubt that under a different system of administration a very large number of beds could be set free for acute cases. Under a centralised system the senile and chronic cases not needing active treatment could be removed to simpler institutions, the staffs could be increased, and specialists, available for consultation or for specialised methods of treatment and diagnosis, appointed, the equipment of all the infirmaries could be improved and brought up to the requirements of modern treatment, and convalescent homes, in which the Poor-Law system is totally deficient, provided. At a conservative estimate at least six or seven thousand beds could be emptied by these means. To set off against this, provision would have to be made for the maternity patients and the acute cases of illness which are at present retained in the sick wards of some workhouses. The number of such cases is however not large, and one may anticipate that after providing for them one would still have at least six thousand beds available in the infirmaries for insured patients. Another point of importance is that beds would be thus set free in all the London infirmaries, and would therefore be pretty uniformly distributed over London, so that the new patients could be treated in convenient proximity to their own homes. This system of centralisation would bring with it two other advantages. There would be one uniform training and one examination for all infirmary probationers, and the amount of experience gained in nursing acute cases would be very considerably increased in many of the infirmaries. It would also be possible to arrange for the attendance of senior medical students to receive clinical teaching in the wards, with advantage both to the patients and to the system of medical education in London.

